# Antiviral Activity of Novel Quinoline Derivatives against Dengue Virus Serotype 2

**DOI:** 10.3390/molecules23030672

**Published:** 2018-03-16

**Authors:** Carolina de la Guardia, David E. Stephens, Hang T. Dang, Mario Quijada, Oleg V. Larionov, Ricardo Lleonart

**Affiliations:** 1 Institute of Scientific Research and High Technology Services (INDICASAT AIP), PO 0843-01103 City of Panama, Panama; cdelaguardia@indicasat.org.pa (C.d.l.G.); MQuijada@indicasat.org.pa (M.Q.); 2 Department of Biotechnology, Acharya Nagarjuna University, Nagarjuna Nagar, Guntur 522510, Andhra Pradesh, India; 3 Department of Chemistry, University of Texas at San Antonio, San Antonio, TX 78249, USA; dstephense@gmail.com (D.E.S.); danghang.hcmut@gmail.com (H.T.D.); Oleg.Larionov@utsa.edu (O.V.L.)

**Keywords:** dengue virus, quinolines, antiviral

## Abstract

Dengue virus causes dengue fever, a debilitating disease with an increasing incidence in many tropical and subtropical territories. So far, there are no effective antivirals licensed to treat this virus. Here we describe the synthesis and antiviral activity evaluation of two compounds based on the quinoline scaffold, which has shown potential for the development of molecules with various biological activities. Two of the tested compounds showed dose-dependent inhibition of dengue virus serotype 2 in the low and sub micromolar range. The compounds **1** and **2** were also able to impair the accumulation of the viral envelope glycoprotein in infected cells, while showing no sign of direct virucidal activity and acting possibly through a mechanism involving the early stages of the infection. The results are congruent with previously reported data showing the potential of quinoline derivatives as a promising scaffold for the development of new antivirals against this important virus.

## 1. Introduction

Dengue fever is a viral re-emergent disease affecting human populations in tropical and subtropical regions. Some estimates indicate that more than 100 countries are affected and more than 390 million people become infected each year, with approximately 20,000 deaths [1]. The disease is caused by dengue virus, resulting in a growing and uncontrolled infection transmitted by mosquitos of the *Aedes* genus. The disease is a serious public health concern, causing a range of clinical manifestations from mild febrile illness in primary infections to the deadly severe dengue in secondary infections [2]. The epidemiological situation is complex in many affected countries due to the intense transmission rate, the coexistence of up to four serotypes, high vector invasiveness, and increased global human mobility.

Dengue virus belongs to the *Flavivirus* genus, from the *Flaviviridae* family, and circulate as four major serotypes which do not induce long-term cross-protection in secondary infections [3]. The development of effective vaccines against dengue virus is technically challenging as it must induce a balanced immune protection against all four serotypes [4,5]. Additionally, there is no specific antiviral therapy approved for patients with dengue disease other than general symptomatic treatment [6]. Since there is no approved antiviral against this virus, the identification of new antivirals is an important endeavor. Although the viremia is short-lived in dengue patients, the use of effective antivirals might be very useful in the case of severe dengue, as high viremia has been shown to correlate with severity [7].

Quinolines are heterocyclic molecules composed of fused benzene and pyridine rings. The quinolines and their derivatives have shown a wide range of biological activities, including antiproliferative [8], antiviral [9], antibacterial [10], antifungal [11], anti-inflammatory [12], and antiparasitic [13]. Members of the quinoline family, such as chloroquine and hydroxychloroquine, have shown antiviral activity against several viruses, such as coronaviruses [9], human immunodeficiency virus [14], and respiratory syncytial virus [15]. Concerning *Flavivirus*, quinoline derivatives have proved active against the Hepatitis C virus [16], West Nile virus [17,18], Japanese Encephalitis virus [19], Zika virus [20], and dengue virus [21].

Since the quinoline scaffold has shown high potential for the development of new molecules with antiviral activity, we decided to synthesize and test several quinoline derivatives and related compounds. Here we describe the synthesis, screening, and characterization of antiviral activity of two novel quinoline derivatives against the dengue virus serotype 2 (DENV2) in vitro.

## 2. Results

Quinoline compounds have shown potential as new scaffolds for the development of drugs with pharmacological activities against various human pathogens, including viruses. In order to investigate the possible antiviral potential of several new quinoline derivatives, we synthesized a number of derivative compounds and tested their ability to impair infection by DENV2 in a medium-throughput assay based on cellular ATP detection.

**Scheme 1:**
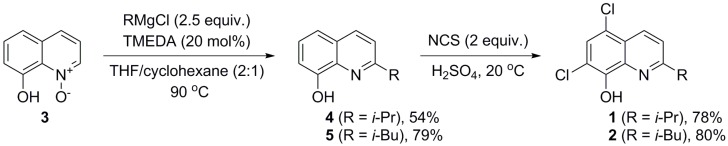
Synthesis of quinoline derivatives, compounds **1** and **2**.

Synthetically, substituted quinolines can be accessed from quinoline or quinoline *N*-oxide precursors. The *N*-oxides are typically prepared by N-oxidation of quinolines [22]. Several methods have recently been developed to prepare substituted quinolines from the unsubstituted precursors [23,24]. We found that a modification of our recently developed *N*,*N*,*N*′,*N*′-tetramethylethylenediamine (TMEDA)-catalyzed synthesis of 2-substituted quinolines [25] provided optimal yields and purity of quinolines **4** and **5** from 8-hydroxyquinoline *N*-oxide (3), although the copper-catalyzed reaction of Grignard reagents [26] gave similar results (Scheme 1). Using the TMEDA-catalyzed reaction, 2-alkylated quinolines **4** and **5** were isolated in 54 and 79% yields, respectively. Subsequent chlorination with *N*-chlorosuccinimide (NCS) under acidic conditions afforded the desired quinolines **1** and **2** in 78 and 80% yields, respectively Scheme 1 and Supplementary Materials). The final purity of both compounds **1** and **2** was verified to be higher than 97%.

A total of 29 compounds, derivatives of the quinoline scaffold and related compounds (Table S1, Supplementary Materials), were tested at the prescreening stage, where both antiviral activity and cytotoxicity were assayed at a single concentration point of 30 µM (data not shown). Most of the tested compounds at the prescreening step either did not show inhibition against DENV2 or displayed evident cytotoxicity (data not shown). Based on the results of the prescreening stage, three compounds were selected for confirmatory tests, where antiviral activity and cytotoxicity were explored using a dose–response approach. 

**Figure 1:**
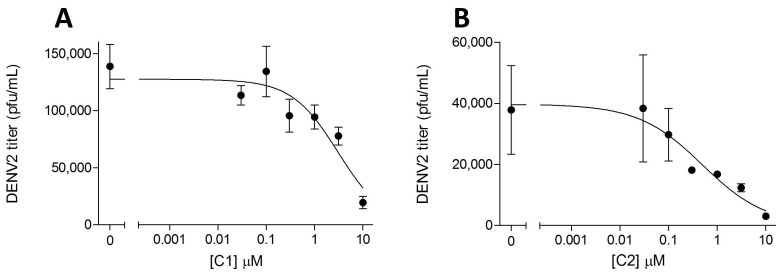
Quinoline derivatives **1** (panel **A**) and **2** (panel **B**) are active against DENV2 in a dose-dependent manner. Cells were infected with DENV2 and treated with several concentrations of either compound **1** (C1), compound **2** (C2), or dimethyl sulfoxide (DMSO) at equivalent concentration, as described in Materials and Methods. Forty-eight hours post infection, supernatants were collected, and viral titers determined by plaque assays. Data is expressed as mean ± standard error of three independent experiments. The curve was obtained by nonlineal regression analysis (four parameters logistic, variable slope model) as implemented in GraphPad Prism (GraphPad Software, La Jolla, CA, USA).

**Figure 2:**
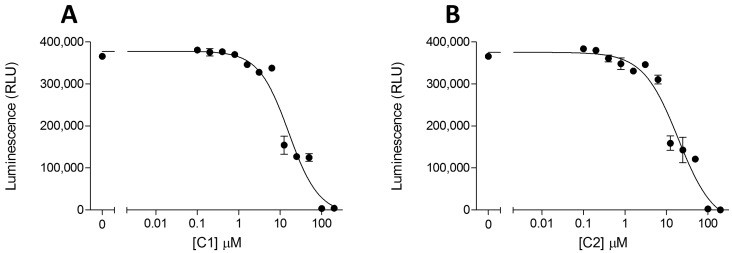
Cytotoxicity data of compounds **1** (panel **A**) and **2** (panel **B**) in Vero cells. Cells were treated with several concentrations of either compound **1** (C1), compound **2** (C2), or DMSO at equivalent concentration, as described in Materials and Methods. After 48 h, cellular adenosine triphosphate (ATP) was detected and measured by chemiluminescence. Data is expressed as mean ± standard error of three independent experiments. The curve was obtained by nonlineal regression analysis (four parameters logistic, variable slope model) as implemented in Graphpad Prism.

In the confirmatory viral yield reduction assay, only compounds **1** and **2** showed consistent inhibitory activity against DENV2 in vitro (Figure 1). Compound **1** showed inhibitory activity with a half maximal inhibitory concentration (IC_50_) of 3.03 µM and a half maximal cytotoxic concentration (CC_50_) of 16.06 µM, for an estimated selectivity index (SI) of 5.30 (Figure 1 and Figure 2). Compound **2** was also active, showing a higher SI of 39.5 (IC_50_: 0.49 µM, CC_50_: 19.39 µM) (Figure 1 and Figure 2). 

**Figure 3:**
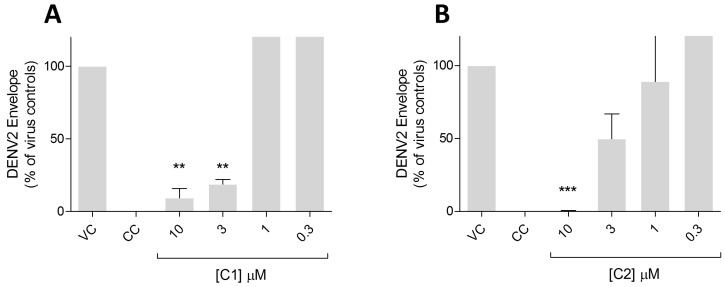
Reduction of viral envelope glycoprotein accumulation in Vero cells infected and treated with compounds **1** (panel **A**) or **2** (panel **B**) or vehicle (DMSO). Cell lysates at 48 h post infection were assayed with Western blot as described in Materials and Methods. The intensity signals were detected using chemiluminescence and normalized to the signal of the loading control at each lane. The normalized value for envelope protein in nontreated, dengue-infected cultures was considered as 100%. VC, virus control, corresponding to infected, non treated cells. CC, cell control, corresponding to non treated, non infected cells. Data is expressed as mean ± standard error of three independent experiments. Statistically different means from the value of the virus control sample are marked as (**) *p* < 0.01 or (***) *p* < 0.001.

To further explore the mechanism of action of these compounds, we measured their capacity to impair the production of the viral envelope glycoprotein; a key protein of the flavivirus life cycle, the E glycoprotein was assessed by Western blot. For this purpose, DENV2-infected cells were treated with several concentrations of compounds **1** or **2**. Forty-eight hours post infection, infected cells treated with compounds showed a significant dose-dependent reduction in the amount of envelope protein detectable by Western blots (Figure 3). The reduction was statistically significant for compound **1** at concentrations of 3 and 10 µM (*p* = 0.0016 and *p* = 0.0054, respectively) and for compound **2** at 10 µM (*p* = 0.0001).

**Figure 4:**
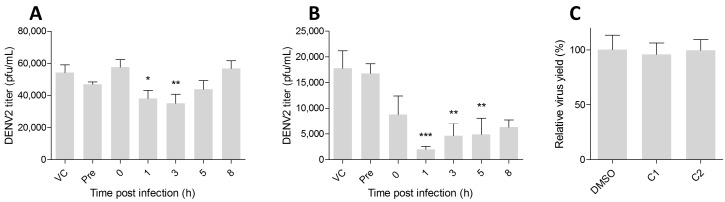
Time-of-addition analysis (**A**,**B**) and effects of active compounds on the virucidal activity assay (**C**). Compound **1** (**A**) or compound **2** (**B**) was added at several time points during DENV2 infection of Vero cells, as described in Materials and Methods. VC: virus control, infected without compound, with a multiplicity of infection (MOI) = 0.5. Pre: compounds were added to cell monolayers for 1 h before infection, and then removed. 0: compounds were incubated with the cells for 1 h, during the virus adsorption, then removed. 1–8: compounds were added at 1–8 h and kept for 48 h post infection. Then supernatants were collected and the titer was determined by plaque assays. For the virucidal activity assay (**C**), DENV2 aliquots containing 10^5^ pfu were incubated with either compound **1**, compound **2**, or vehicle for 1 h. Then the virus was diluted in maintenance medium and the viral titer determined by plaque assays. Data is expressed as mean ± standard error of three independent experiments. Statistical analysis was done as detailed in Materials and Methods. Statistically different means from the value of the virus control sample are marked as (*) *p* < 0.05, (**) *p* < 0.01 or (***) *p* < 0.001.

To evaluate the possible mechanism of action of these compounds, we performed viral yield reduction assays varying the time of their addition to the infected cultures. The compounds were added at the pre-infection period, during the infection, or at different time points after the infection (1 h, 3 h, 5 h, or 8 h). Then, infected cultures were maintained, and supernatants collected at 48 h post-infection. The determination of viral titers in these supernatants may indicate at which stage of the viral life cycle the compounds may be acting. Compound **1** showed maximal impairment of the DENV2 infection when present from 1 h post-infection, losing effect gradually when added at later time points (Figure 4A). The effect of this compound was not significant when added at the pre-infection period or during the adsorption phase only, suggesting that its action is not due to virucidal activity. While not significantly different from the virus control, compound **1** did show a small effect on virus infectivity when added only during the adsorption phase, suggesting some possible virucidal activity or impairment of the attachment/entry process. Compound **2** also significantly reduced the viral yield when present from the 1 h time point (Figure 4B). Similarly, its effect was not noticeable when added at pre-infection or during the viral adsorption. These results suggest that both compounds act, at least, affecting the early events of the virus life cycle.

Since certain active antiviral molecules may act upon direct contact with virions, we wanted to further evaluate if compounds **1** and **2** had virucidal activity. Incubation of the DENV2 virions for one hour with 3 µM of compound **1** or **2**, or the corresponding vehicle (DMSO), prior to adsorption to the monolayers did not show any significant reduction in the viral titer (Figure 4C). This result suggests that the antiviral effect of these molecules is not due to direct virucidal activity. 

## 3. Discussion

The quinoline moiety is commonly found in natural products, showing a wide variety of biological activities. Based on this fact, many synthetic derivatives have been synthesized and examined for pharmacological activities and properties. Currently there are several approved drugs that contain quinoline structural cores, such as bedaquiline and the semisynthetic irinotecan, for drug resistant tuberculosis and colorectal cancer, respectively [27,28].

Here we have shown that two novel derivatives of the quinoline scaffold have antiviral activity against DENV2 in the low micromolar range. These results are encouraging to further characterize and improve the biological activity profile of new synthetic variants. Both compounds **1** and **2** also showed a significant, dose-dependent effect on the production of the viral envelope glycoprotein, congruent with the antiviral activity shown in the virus yield reduction assay. This effect is commonly observed in other molecules with antiviral activity against dengue virus. Our data suggests that this antiviral activity is not due to direct virucidal activity, and that compounds seem to be acting at an early stage of the viral life cycle. More experimental data is required to precisely describe the targets of these compounds. 

Our results are consistent with other published data showing the potential of this scaffold for the development of new antivirals. Several reports have described that other derivatives of the 8-hydroxyquinoline scaffold are potent inhibitors of several flaviviruses, including DENV2 [21] and West Nile virus [18,29]. Amodiaquine, a derivative of 4-aminoquinoline, was recently described to have activity against DENV2 [30]. Additionally, the quinoline derivative compound RG7109 was found to be a potent inhibitor of the NS5B polymerase from the Hepatitis C virus, a flavivirus that affects 150 million people worldwide [16].

Certain quinoline derivatives have shown diverse biological activities. For example, chloroquine, a 9-aminoquinoline with antimalarial activity, was also shown to inhibit flavivirus replication [31,32,33] as well as that of other viruses such as influenza [34], HIV [35], and coronavirus [9].

Both active molecules in our study are structurally related to chlorquinaldol (unique chemical structure identifier CID: 6301). According to the PubChem database (https://pubchem.ncbi.nlm.nih.gov/compound/Chlorquinaldol), this compound has been found to be active against Marburg virus, Venezuelan equine encephalitis virus, and foot-and-mouth disease virus [36]. However, in our assay, chlorquinaldol was not active against DENV2 (data not shown), suggesting that the modifications shown in **1** and **2** are critical for the observed antiviral activity. Additionally, several other compounds tested in our initial screening with different 2-substitutions were also inactive, further emphasizing the role of the alkyl groups shown in compounds **1** and **2** (data not shown). Similarly, 8-hydroxyquinoline (8-HQ) and some related derivatives, including 5,7-dichloro-8-HQ, were reported to have activity against Rous sarcoma virus and herpes simplex virus due to their inhibition of the RNA-dependent DNA polymerase [37]. Other investigations have shown that 8-HQ derivatives are strong in vitro inhibitors of the West Nile virus protease [18] as well as the DENV2 protease NS2B/NS3 in the sub micromolar range [21]. 

We have shown here two novel quinoline derivatives that are active against DENV2 in a dose-dependent manner. These results are important as a starting point for further elucidation of the precise mechanisms of the antiviral activity and to gain the required knowledge to further develop new, more potent and safe drugs to reduce the infections caused by this relevant pathogen.

## 4. Materials and Methods 

### 4.1. Cells, Viruses and Compounds

Vero cells and dengue virus, serotype 2, were a kind gift from Dr. Sandra López-Vergès, Instituto Conmemorativo Gorgas de Estudios de la Salud, Panamá. Vero cells were grown in Minimal Essential Medium (MEM) supplemented with 10% fetal bovine serum (FBS). The FBS was reduced to 1% in the maintenance medium that was used in virus assay. Evaluated compounds were synthesized as described below. For antiviral assays, the lyophilized synthetic compounds were resuspended in dimethyl sulfoxide (DMSO, Sigma-Aldrich, St. Louis, MO, USA). 

### 4.2. Synthetic Procedures

Compound synthesis and purification: Anhydrous tetrahydrofuran was collected under argon from a solvent purification system (LC Technology Solutions, Salisbury, MA 01952, USA), having been passed through two columns packed with molecular sieves. 8-Hydroxyquinoline *N*-oxide (**3**) was prepared according to the literature procedure [22]. *N*,*N*,*N*′,*N*′-Tetramethylethylenediamine (TMEDA) was freshly distilled from calcium hydride. All other chemicals were used as commercially available. All reactions were conducted with continuous magnetic stirring under an atmosphere of argon in oven-dried glassware. Reactions were monitored by ^1^H NMR or by TLC on silica-gel-coated glass plates (Merck Kieselgel 60 F254, Merck KGaA, Darmstadt, Germany) until deemed complete. Plates were visualized under ultraviolet light (254 nm) and by staining with ceric ammonium molybdate (CAM) or potassium permanganate. Column chromatography was performed using a CombiFlash Rf-200 (Teledyne-Isco, Lincoln, NE, USA) automated flash chromatography system.

*2-Isopropylquinolin-8-ol* (**4**). To a solution of 8-hydroxyquinoline *N*-oxide (**3**) (161.2 mg, 1 mmol) and *N*,*N*,*N*′,*N*′-tetramethylethylenediamine (30 mL, 0.2 mmol), in a mixture of THF (2 mL) and cyclohexane (1 mL) in a pressure tube, was added isopropylmagnesium chloride (2M solution in THF, 1.25 mL, 2.5 mmol). The reaction was heated at 90 °C for 16 h, after which it was cooled, quenched with NH_4_Cl (4 mL), and extracted with EtOAc (3 × 15 mL). The organic phase was combined, dried over anhydrous Na_2_SO_4_, and concentrated under reduced pressure. The crude product was purified by preparative TLC (EtOAc/hexane, 1:100 *v*/*v*) to give 2-isopropylquinolin-8-ol (**4**) (102 mg, 54%) as a light-green solid.—mp 99–103 °C.—^1^H NMR (500 MHz, CDCl_3_): 8.07 (1 H, d, *J* = 8.5 Hz), 7.42–7.33 (1 H, m), 7.29 (1 H, d, *J* = 8.1 Hz), 7.14 (1 H, d, *J* = 7.5 Hz), 3.24 (1 H, m), 1.40 (4 H, d, *J* = 6.9 Hz) ppm.—^13^C NMR (125 MHz, CDCl_3_): 165.5, 152.0, 136.5, 136.1, 126.8, 120.9, 117.6, 114.0, 109.7, 36.7, 22.5 ppm.—IR: 3185, 2963, 2930, 2871, 1599, 1572, 1505, 1458, 1386, 1226, 1197, 837.

*5*,*7-Dichloro-2-isopropylquinolin-8-ol* (**1**). A solution of 2-isopropylquinolin-8-ol (**4**) (66 mg, 0.35 mmol) and *N*-chlorosuccinimide (93 mg, 0.7 mmol) in 96% sulfuric acid (1.3 mL) was stirred at room temperature for 16 h. The reaction mixture was neutralized with a saturated solution of sodium carbonate and extracted with EtOAc (3 × 10 mL). The organic phase was combined, dried over anhydrous Na_2_SO_4_, and concentrated under reduced pressure. The crude product was purified by flash chromatography on silica gel (EtOAc/hexane, 1:100 *v*/*v*) to give 5,7-dichloro-2-isopropylquinolin-8-ol (**1**) (70 mg, 78%) as a light-brown solid.—mp 116–120 °C.—^1^H NMR (500 MHz, CDCl_3_): 8.47–8.23 (1 H, m), 7.54–7.32 (2 H, m), 3.38–3.12 (1 H, m), 1.39 (5 H, d, *J* = 7.0 Hz) ppm.—^13^C NMR (125 MHz, CDCl_3_): 167.4, 147.3, 138.0, 133.9, 127.3, 123.6, 121.6, 120.7, 115.0, 36.6, 22.4 ppm.—IR: 3360, 2964, 2928, 2871, 1594, 1497, 1482, 1450, 1410, 1349, 1309, 1196, 1082, 832.—HRMS calcd for C_12_H_11_Cl_2_NO: 256.0290, found 256.0290 [M + H^+^]. 

*2-Isobutylquinolin-8-ol* (**5**). To a solution of 8-hydroxyquinoline *N*-oxide (**3**) (81 mg, 0.5 mmol) and *N*,*N*,*N*′,*N*′-tetramethylethylenediamine (150 mL, 0.1 mmol), in a mixture of THF (1 mL) and cyclohexane (0.5 mL) in a pressure tube, was added isobutylmagnesium chloride (2M solution in THF, 0.625 mL, 1.25 mmol). The reaction was heated at 90 °C for 16 h, after which it was cooled, quenched with NH_4_Cl (2 mL), and extracted with EtOAc (3 × 10 mL). The organic phase was combined, dried over anhydrous Na_2_SO_4_, and concentrated under reduced pressure. The crude product was purified by preparative TLC (EtOAc/hexane, 1:100 *v*/*v*) to give 2-isobutylquinolin-8-ol (**5**) (80 mg, 79%) as a brown liquid.—^1^H NMR (500 MHz, CDCl_3_): 8.04 (1 H, d, *J* = 8.4 Hz), 7.39 (1 H, t, *J* = 7.9 Hz), 7.31–7.27 (2 H, m), 7.15 (1 H, dd, *J* = 7.5, 1.0 Hz), 2.83 (2 H, d, *J* = 7.3 Hz), 2.24 (1 H, dt, *J* = 13.6, 6.9 Hz), 0.98 (6 H, d, *J* = 6.6 Hz) ppm.—^13^C NMR (125 MHz, CDCl_3_): 160.2, 151.9, 137.8, 136.1, 126.9, 126.8, 123.0, 117.6, 109.7, 47.9, 29.3, 22.7 ppm.—IR: 3383, 3052, 2954, 2868, 1602, 1571, 1506, 1470, 1367, 1324, 1250, 1200.

*5*,*7-Dichloro-2-isobutylquinolin-8-ol* (**2**). A solution of 2-isobutylquinolin-8-ol (**5**) (80 mg, 0.4 mmol) *N*-chlorosuccinimide (107 mg, 0.8 mmol) in 96% sulfuric acid (1.5 mL) was stirred at room temperature for 16 h. The reaction mixture was neutralized with saturated sodium carbonate and extracted with EtOAc (3 × 10 mL). The organic phase was combined, dried over anhydrous Na_2_SO_4_, and concentrated under reduced pressure. The crude product was purified by flash chromatography on silica gel (EtOAc/hexane, 1:100 *v*/*v*) to give 5,7-dichloro-2-isobutylquinolin-8-ol (**2**) (86 mg, 80%) as a light-yellow solid.—mp 52–55 °C.—^1^H NMR (500 MHz, CDCl_3_): 8.71–8.11 (1 H, m), 7.40–7.35 (1 H, m), 7.40–7.35 (1 H, m), 2.93–2.79 (1 H, m), 2.29–2.12 (1 H, m), 0.96 (1 H, dd, *J* = 6.6, 1.1 Hz) ppm.—^13^C NMR (125 MHz, CDCl_3_): 162.1, 147.3, 138.2, 133.5, 127.3, 123.7, 123.4, 120.7, 115.0, 47.6, 29.3, 22.6 ppm.—IR: 3360, 2955, 2868, 1595, 1496, 1449, 1391, 1349, 1323, 1253, 1194, 1145.—HRMS calcd for C_13_H_13_Cl_2_NO: 270.0447, found 270.0446 [M + H^+^].

### 4.3. Primary Screening Antiviral Activity Assay

Cytopathic effect caused by DENV2 was quantified in a medium- to high-throughput assay by measuring cellular ATP levels. A commercially available kit (CellTiter-Glo, Promega Corporation, Madison, WI, USA) was used as recommended by the manufacturer. Assay conditions were optimized for the best signal-to-noise ratio under our lab conditions. Optimized parameters included 10^4^ Vero cells per well (96-well plates), and a multiplicity of infection (MOI) of 3. Hits were defined as compounds producing a signal above the threshold, defined as the mean of untreated, infected cells plus three standard deviations.

### 4.4. Confirmatory Virus Yield Reduction Assay

Vero cells (8 × 10^4^ cells, 500 µL) were seeded in 24-well plates in complete medium and incubated for 18 h at 37 °C and 5% CO_2_ atmosphere. Monolayers were then washed with phosphate-buffered saline (PBS) and infected with DENV2, MOI of 0.5, in 100 µL at 37 °C with occasional agitation. After 1 h, the virus was aspirated, and cells were washed once with PBS and replenished with 500 µL of maintenance medium containing compounds at several concentrations or vehicle. Forty-eight hours later, the supernatant was recovered and virus yield were quantified by plaque assay. For plaque assays, 2 × 10^5^ cells were seeded per well in 12-well plates in complete medium and incubated for 24 h. Then, monolayers were washed with PBS and infected with several dilutions of the collected supernatants, using 150 µL per well for 1 h at 37 °C with occasional agitation. Then, the infected cells were covered with an overlay containing maintenance medium plus 1% SeaPlaque agarose (Lonza Group Ltd., Basel, Switzerland). After incubation for 5 days, formaldehyde 3.7% in PBS was added for 2 h at room temperature, the overlay plug discarded, and cells stained with 100 µL of crystal violet in 30% ethanol for 1 min. After washing in running water, plates were dried and plaques counted. The reduction in virus yield was expressed as the reduction in virus titers in compound-treated cells as compared to nontreated, virus-only controls.

### 4.5. Cytotoxicity Assay

Cytotoxicity of compounds was assessed by ATP detection using a commercially available kit, as per manufacturer instruction (CellTiter-Glo, Promega, Madison, WI, USA). Briefly, 10^4^ Vero cells were seeded per well in a 96-well plate using 100 µL of complete medium. After 18 h, medium was replaced with several concentrations of compounds in maintenance medium and incubated further for 48 h. Then, 100 µL of freshly reconstituted ATP detection reagent was added to each well and the plate carefully agitated for 2 min; cell lysates were transferred to opaque white plates (Thermo Fisher Scientific, Waltham, MA, USA, Cat. No. 236105) and the luminescence signal was detected after 10 min using a multidetection microplate reader (Synergy HT, Biotek, Winooski, VT, USA). Cytotoxicity was expressed as the reduction of luminescent signal in compound-treated cells as compared to nontreated cells. 

### 4.6. Western Blot Assay

Cell lysates were prepared from infected and compound-treated cells, 48 h post infection. Twenty-five micrograms of total protein were loaded into SDS-PAGE gels under nonreducing conditions. After electrophoresis, proteins were transferred to PVDF membrane (Amersham™, Amersham, UK) and blocked with 3% (*w*/*v*) BSA in Tris-buffered saline (TBS) containing 0.05% (*v*/*v*) Tween-20. After blocking, the membrane was incubated overnight at 4 °C with anti-envelope primary antibody (4G2, Absolute Antibody, Redcar, UK), then with horseradish peroxidase (HRP)-conjugated rabbit anti-mouse and signal developed using chemiluminescence. Blots were stripped and re-probed with anti-ERK2 (Santa Cruz Biotechnology, Dallas, TX, USA) as a loading control. The signal corresponding to the envelope protein was normalized against the loading control using Image Studio Lite v5.2 (LI-COR, Lincoln, NE, USA) as recommended by the manufacturer. 

### 4.7. Time-of-Addition Analysis

Vero cells were seeded and infected as described in the confirmatory virus yield reduction assay, except that compounds were added at several time points at 3 µM. For preincubation, compounds were added 1 h before infection and removed just before virus adsorption. For incubation during infection (timepoint “0”), compounds were added and incubated with cells for 1 h, exactly during the viral adsorption period, then removed and cells washed. For post-infection timepoints, compounds were added at 1, 3, 5, and 8 h in maintenance medium and kept until 48 h post infection, when supernatants were collected and virus titer determined by plaque assays. 

### 4.8. Virucidal Activity Assay

Compounds at 3 µM, or the corresponding vehicle (DMSO at equivalent concentration), were incubated with 10^5^ pfu of DENV2 for 1 h at 37 °C in maintenance medium. Then, the samples were diluted in maintenance medium and tested in a plaque assay to determine viral survival after treatment. 

### 4.9. Statistical Analysis

Data is presented as mean and standard error of the mean from three independent experiments. Means of each group were compared to virus controls (100%) using one-sample *t*-tests as implemented in Graphpad Prism v5.0. Statistically significant difference was considered if *p* < 0.05. Half maximal inhibitory concentrations (IC_50_) and half maximal cytotoxic concentrations (CC_50_) were calculated by fitting a nonlineal regression model to the data (four parameters logistic, variable slope model) as implemented in Graphpad Prism. Selectivity index (SI) was calculated as the ratio CC_50_/IC_50_.

## 5. Conclusions

Here we have described two novel quinoline derivatives that are very active against dengue virus serotype 2. These compounds are not virucidal and appear to act at an early stage of the virus life cycle, reducing the intracellular production of the envelope glycoprotein and the yield of infective virions in treated and infected cells. These results are important start points to develop new and safer antivirals against this important virus.

## Supplementary Materials

The following are available online, Figure S1: Spectral data of compounds shown in this study, Table S1: Structures of all compounds tested for virucidal activity in this study.
