# Supplementary material for: Antiviral Activity of Novel Quinoline Derivatives against Dengue Virus Serotype 2
Source: Molecules. 2018 Mar 16;23(3):672. doi: 10.3390/molecules23030672 (PMC5997395; doi:10.3390/molecules23030672)
Supplement: Supplementary file 1 [file molecules-23-00672-s001.pdf]

Supplementary Material

Communication

Antiviral Activity of Novel Quinoline Derivatives against Dengue Virus Serotype 2

Carolina de la Guardia [1,2], David E. Stephens [3], Hang T. Dang [3], Mario Quijada [1], Oleg V. Larionov [3] and Ricardo Lleonart [1,*]

[1] Institute of Scientific Research and High Technology Services (INDICASAT AIP), PO 0843-01103, City of Panama, Panama; cdelaguardia@indicasat.org.pa (C.d.l.G.); MQuijada@indicasat.org.pa (M.Q.)
[2] Department of Biotechnology, Acharya Nagarjuna University, Nagarjuna Nagar, Guntur - 522 510, Andhra Pradesh, India
[3] Department of Chemistry, University of Texas at San Antonio, San Antonio, TX 78249, USA; dstephense@gmail.com (D.E.S.); danghang.hcmut@gmail.com (H.T.D.); Oleg.Larionov@utsa.edu (O.V.L.)
* Correspondence: rlleonart@indicasat.org.pa; Tel.: +507-517-0740

Figure S1: Spectra Data of Compounds.

2-Isopropylquinolin-8-ol (4)

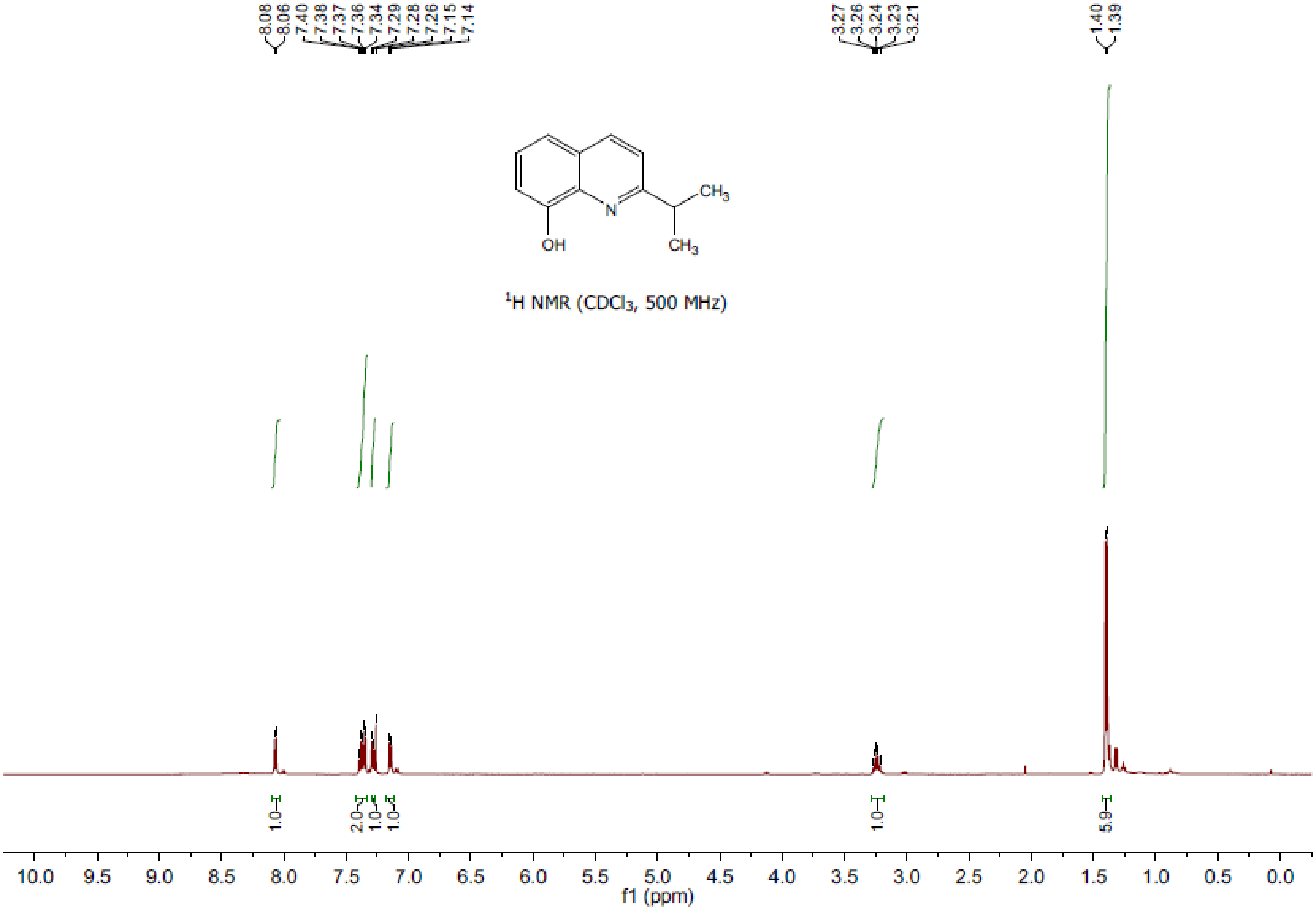

2-Isopropylquinolin-8-ol (4)

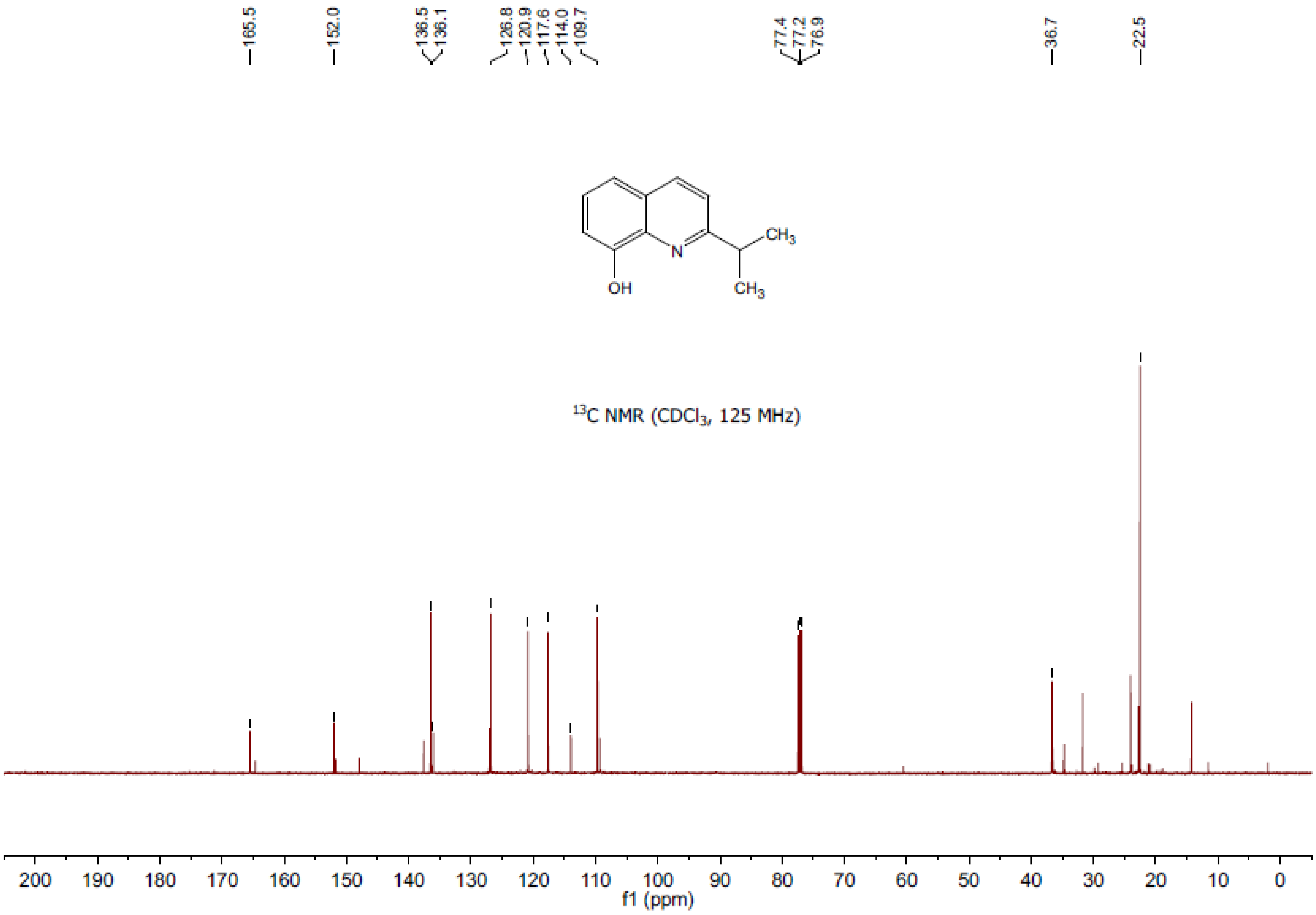

5,7-Dichloro-2-isopropylquinolin-8-ol (1)

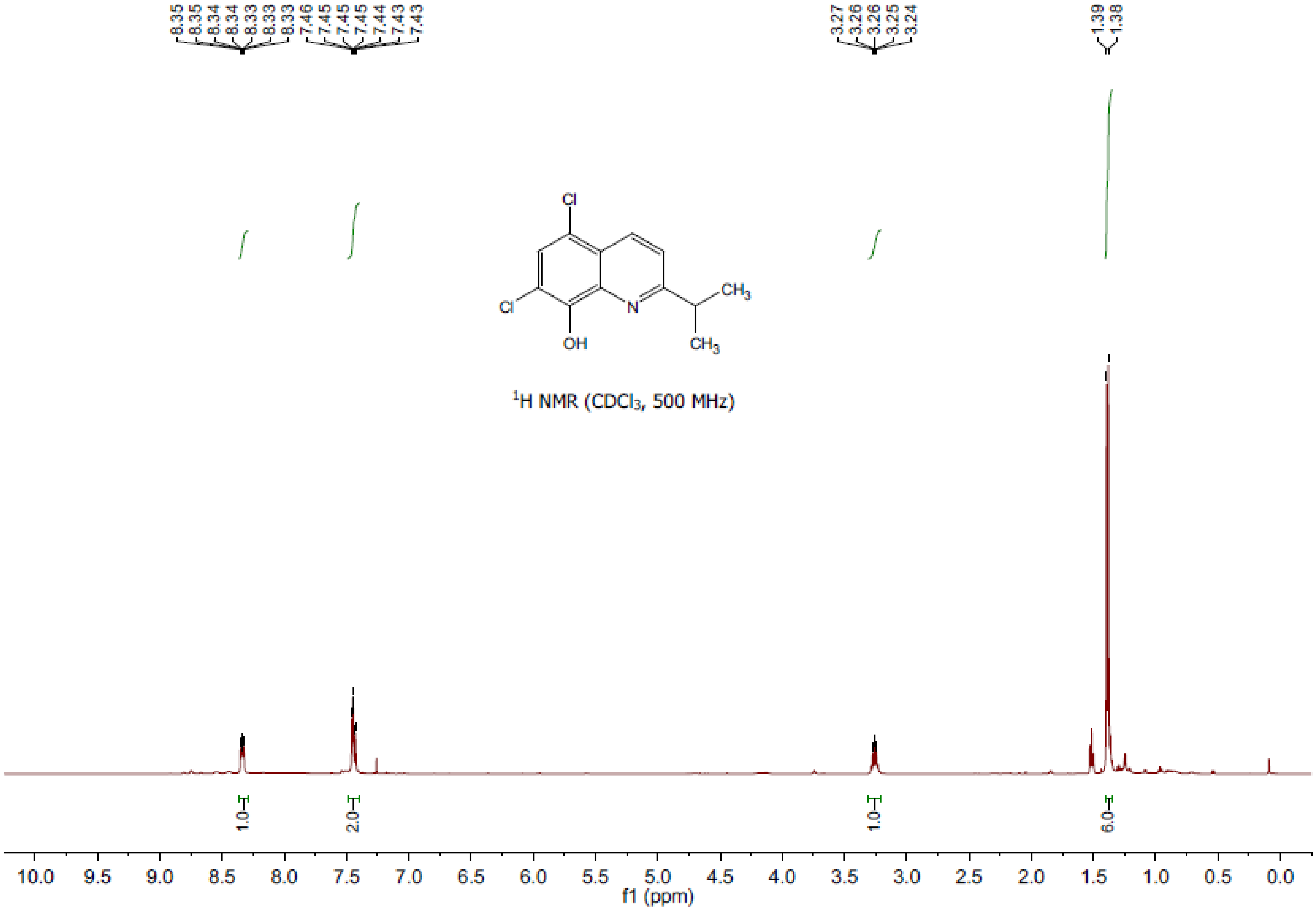

5,7-Dichloro-2-isopropylquinolin-8-ol (1)

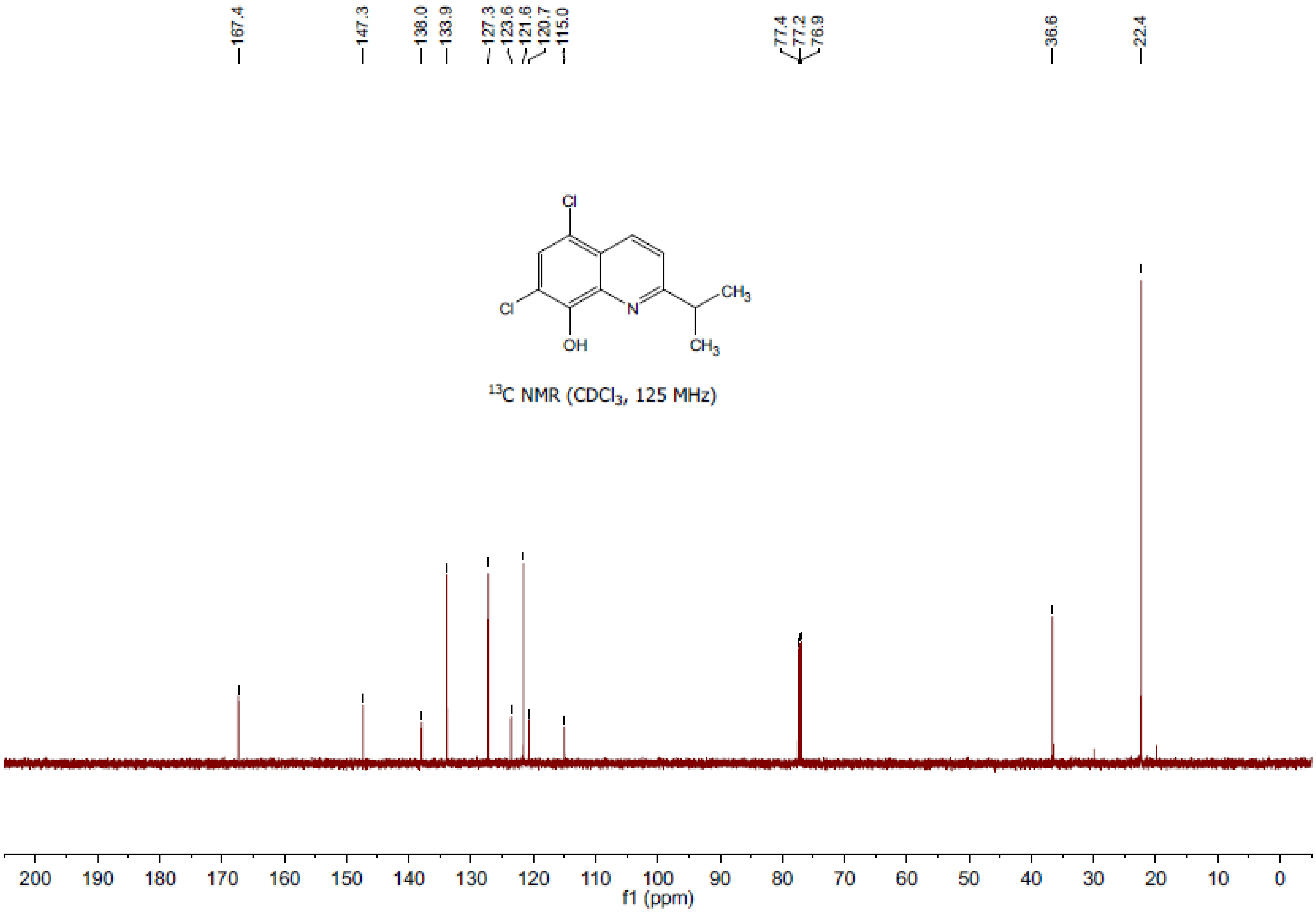

167.4
147.3
138.0
133.9
127.3
123.6
121.6
120.7
115.0
77.4
77.2
76.9
36.6
22.4
Cl
Cl
N
OH
CH3
CH3
13C NMR (CDCl3, 125 MHz)
200 190 180 170 160 150 140 130 120 110 100 90 80 70 60 50 40 30 20 10 0
f1 (ppm)

2-Isobutylquinolin-8-ol (5)

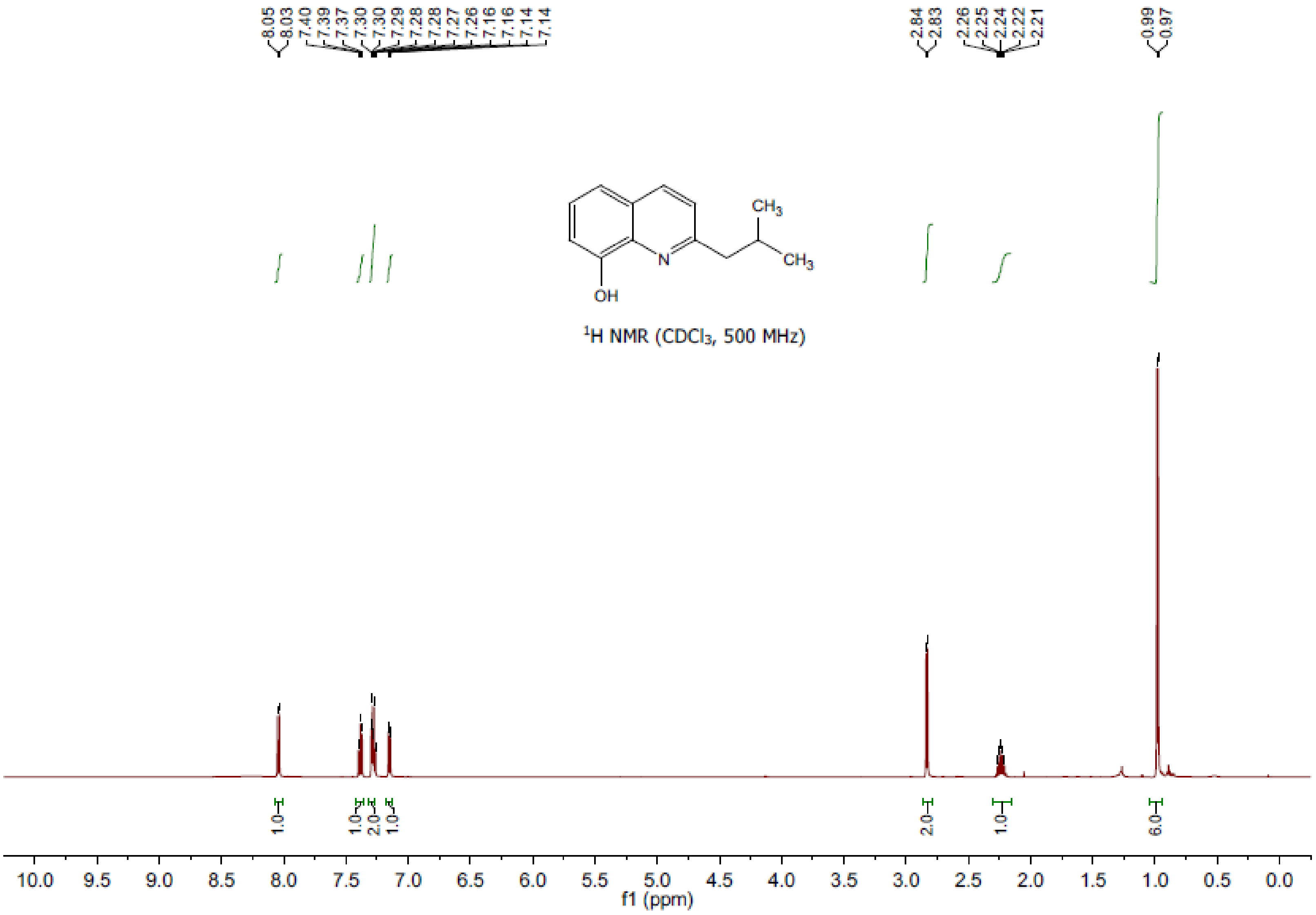

2-Isobutylquinolin-8-ol (5)

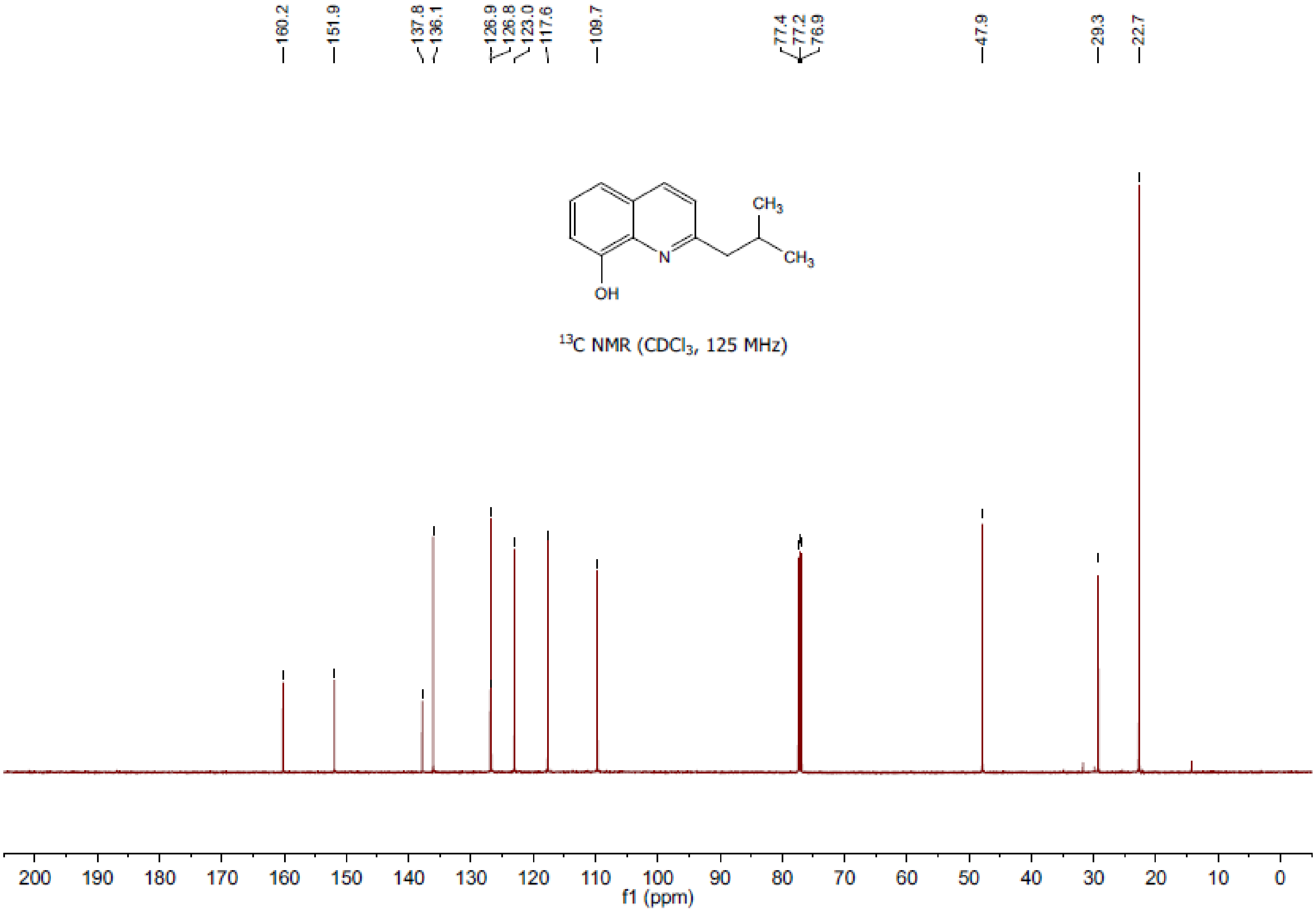
160.2
151.9
137.8
136.1
126.9
126.8
123.0
117.6
109.7
77.4
77.2
76.9
47.9
29.3
22.7
CH3
CH3
N
OH
13C NMR (CDCl3, 125 MHz)
200 190 180 170 160 150 140 130 120 110 100 90 80 70 60 50 40 30 20 10 0
f1 (ppm)

5,7-Dichloro-2-isobutylquinolin-8-ol (2)

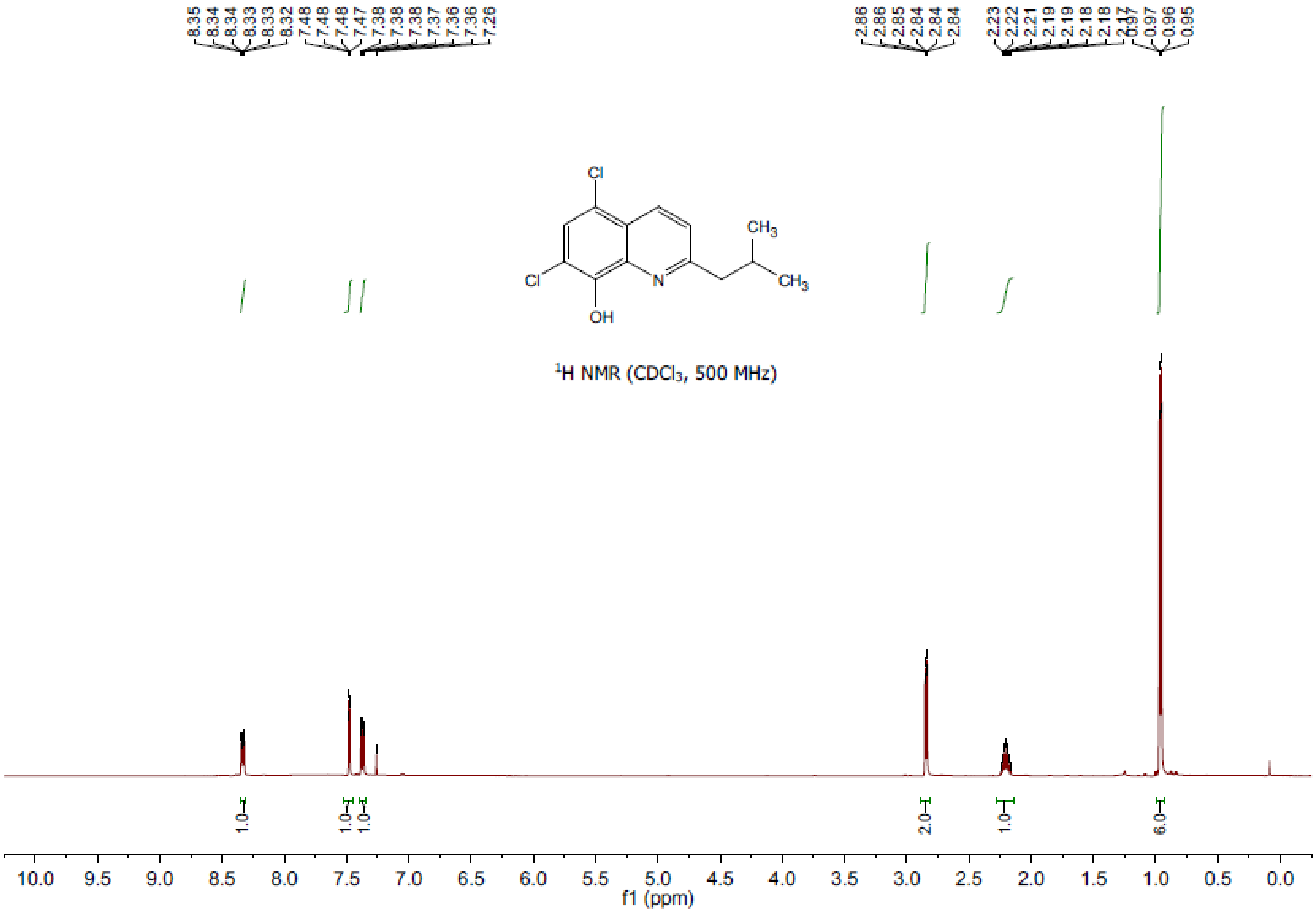

5,7-Dichloro-2-isobutylquinolin-8-ol (2)

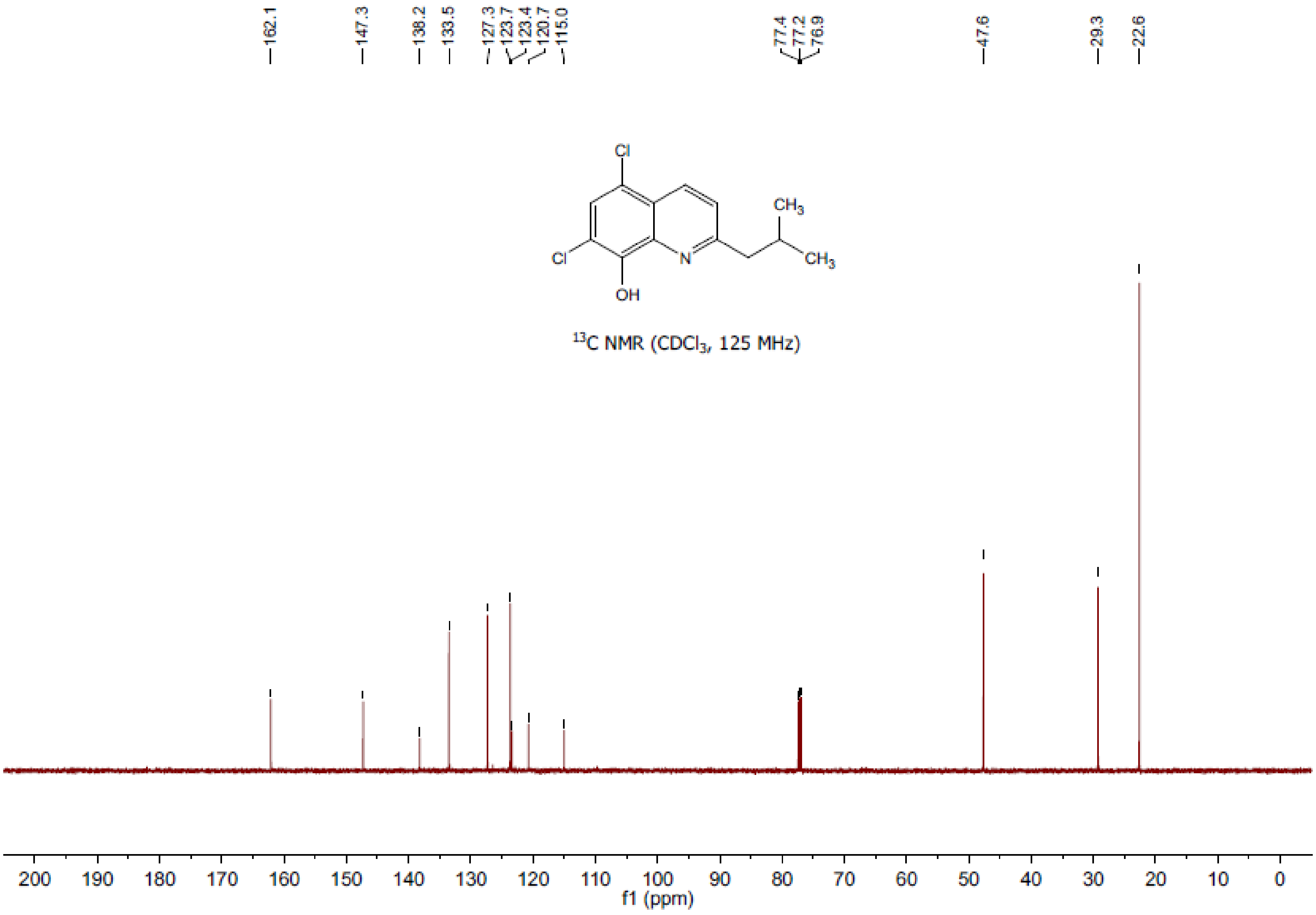
162.1
147.3
138.2
133.5
127.3
123.7
123.4
120.7
115.0
77.4
77.2
76.9
47.6
29.3
22.6
Cl
Cl
OH
N
CH3
CH3
13C NMR (CDCl3, 125 MHz)
200 190 180 170 160 150 140 130 120 110 100 90 80 70 60 50 40 30 20 10 0
f1 (ppm)

molecules

Table S1. Structures of all compounds tested for virucidal activity in this study.

Compound	Structure	Compound	Structure
**1**	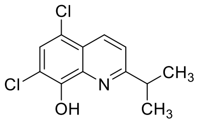	**57**	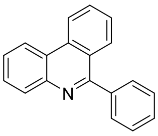
**2**	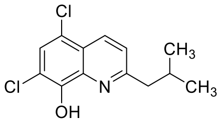	**47**	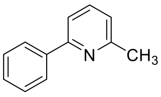
**6**	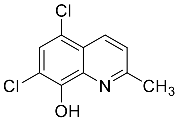	**36**	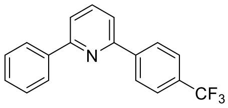
**7**	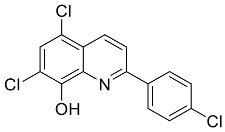	**8**	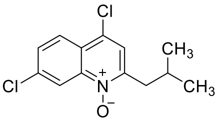
**8**	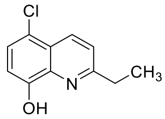	**10**	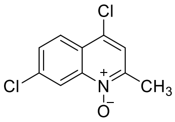
**9**	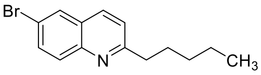	**39**	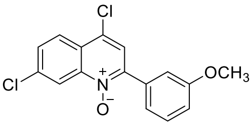
**10**	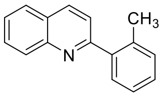	**45**	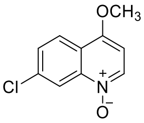
**11**	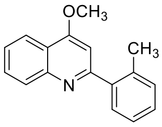	**22**	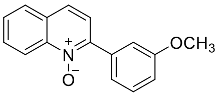
**12**	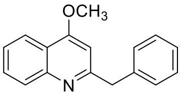	**43**	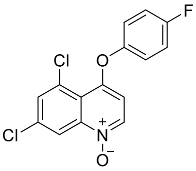
**13**	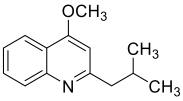	**25**	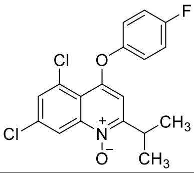
**14**	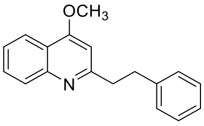	**52**	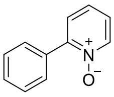

**15**	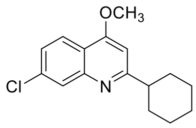	**24**	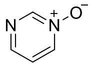
**16**	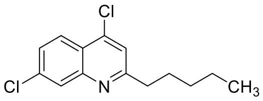	**13**	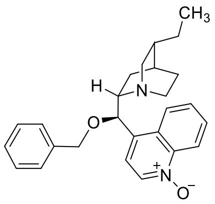
**17**	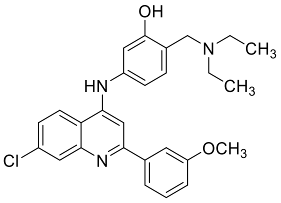	**31**	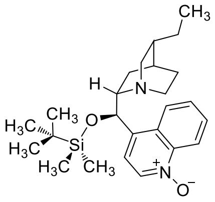
**18**	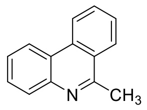		
